# V-Set and immunoglobulin domain containing (VSIG) proteins as emerging immune checkpoint targets for cancer immunotherapy

**DOI:** 10.3389/fimmu.2022.938470

**Published:** 2022-09-15

**Authors:** Xia Zhou, Sohail Khan, Dabing Huang, Lu Li

**Affiliations:** ^1^ Department of Oncology, The First Affiliated Hospital of University of Science and Technology of China (USTC), Division of Life Sciences and Medicine, University of Science and Technology of China, Hefei, China; ^2^ Division of Life Sciences and Medicine, University of Science and Technology of China, Hefei, China

**Keywords:** immune checkpoint, VSIG4/CRIg, VSIG, TIGIT, cancer immunotherapy, antitumor T-cell response, coinhibitory receptor

## Abstract

The development of immune checkpoint inhibitors is becoming a promising approach to fight cancers. Antibodies targeting immune checkpoint proteins such as CTLA-4 and PD-1 can reinvigorate endogenous antitumor T-cell responses and bring durable advantages to several malignancies. However, only a small subset of patients benefit from these checkpoint inhibitors. Identification of new immune checkpoints with the aim of combination blockade of multiple immune inhibitory pathways is becoming necessary to improve efficiency. Recently, several B7 family-related proteins, TIGIT, VSIG4, and VSIG3, which belong to the VSIG family, have attracted substantial attention as coinhibitory receptors during T-cell activation. By interacting with their corresponding ligands, these VSIG proteins inhibit T-cell responses and maintain an immune suppressive microenvironment in tumors. These results indicated that VSIG family members are becoming putative immune checkpoints in cancer immunotherapy. In this review, we summarized the function of each VSIG protein in regulating immune responses and in tumor progression, thus providing an overview of our current understanding of VSIG family members.

## Introduction

Immune checkpoint receptors are membrane molecules that can modulate lymphocyte activation upon encoding their cognate ligands on antigen-presenting cells or target cells. They play an essential role in controlling excessive immune responses by transmitting a stop signal to attenuate T-cell activation and maintain immune homeostasis. However, tumors always take advantage of these inhibitory pathways to escape attack from antitumor immune cells (1, 2). Various malignancies are found to confer an overall immunosuppressive tumor microenvironment by upregulating the expression of immune checkpoint receptors and their ligands. To unleash effector T-cell responses and enhance endogenous antitumor activity, therapies targeting these immunoregulatory proteins are becoming an encouraging approach. The most successful immune checkpoint blockade (ICB) therapy is anti-PD-1/PD-L1, which has been shown to confer therapeutic advantages for a variety of cancers, such as non-small cell lung carcinoma (NSCLC), malignant melanoma, kidney cancer, and liver cancer (3–8). Another well-studied immune checkpoint is cytotoxic T-lymphocyte-associated protein 4 (CTLA-4) (1). The first immune checkpoint inhibitor (ipilimumab) targeting CTLA-4 was approved in 2011 by the Food and Drug Administration (FDA) and has been demonstrated to control tumor growth and prolong survival in melanoma (9–11). Since then, the application of ICBs has brought a groundbreaking paradigm shift in cancer treatment, particularly for advanced-stage cancers (9, 12–14). With the growing research interest in cancer immunotherapy, many new checkpoints have been identified and extensively studied in recent years, such as TIM3, TIGIT, VISTA, LAG-3, BTLA, B7-H3, B7-H4, and B7-H5 (15–19). Most of them belong to the B7 family (VISTA, B7-H3, B7-H4, B7-H5), which is characterized by typical extracellular IgV-like and IgC-like domains and is categorized as the immunoglobulin superfamily (IgSF) (20). These proteins can function as coinhibitory receptors to deliver negative signaling towards T cells upon TCR engagement, therefore inhibiting T-cell activation, expansion, and functional polarization. Recently, the V-Set and immunoglobulin (Ig) domain-containing (VSIG) family, which also belongs to IgSF and exhibits structural similarity with the B7 family proteins, has been increasingly recognized as a potential immune checkpoint contributing to tumor evasion. This family is currently comprised of eight members, including VSIG1, VSIG2, VSIG3, VSIG4, VSIG8, VSIG9, VSIG10, and VSIG10 L, which are all type I transmembrane proteins that are expressed by a variety of both immune and nonimmune cells, and many possess immunosuppressive properties. For example, VSIG3, a ligand of VISTA, is essential for its role in T-cell suppression (21, 22). Another member, VSIG4, is also well known for its potent ability to suppress T-cell responses (23). A star member of the VSIG family is VSIG9, also known as TIGIT, which has emerged as a promising cancer therapeutic target due to its apparent function in limiting antitumor T-cell and NK-cell responses (24–26). These studies suggested that the members of the VSIG family could be potent candidates for developing novel ICB therapies. However, there is still a range of related proteins in this family that have yet to be studied extensively, and the mechanism whereby VSIG family proteins inhibit immune responses is not fully understood. To attract more attention to this family, this review aims to introduce VSIG family members and their role in regulating the T-cell response in cancers.

## Overview of the VSIG family

The discovery of the VSIG protein family dates back to the 1990s. The first identified member of the VSIG family is VSIG2, initially called CTX. This gene was first cloned by Chrétien and colleagues from the cortical thymocyte of Xenopus in 1996 (27). The homolog gene of *VSIG2* in chickens, mice, and humans was cloned two years later (28). It is located on chromosome 11, 11q24.2. The second member, *VSIG4*, was cloned and found to be localized in the pericentromeric region of the human X chromosome (29). Since then, other members showing sequence similarities with these identified VSIG proteins have been discovered, including VSIG1, VSIG3, VSIG8, VSIG9 (widely known as TIGIT), VSIG10, and VSIG10 L. The corresponding genes of those VSIG family members are present on different chromosomes in humans, with the exception of *VSIG1*, which is also present on the X chromosome at a different position from *VSIG4* (Xq22.3 and Xq12, respectively). Of these eight members, only the structures of VSIG4 and TIGIT (RCSB-PDB ID: 5IMK and 3Q0H, respectively) were experimentally resolved through X-ray crystallization, while the rest had computationally predicted structures available. From these data, VSIG family members have been shown to have either an IgV domain, an IgC2 domain, or both (23, 26, 28–33). The IgV domain is shared by all members except for VSIG10 and VSIG10 L (UniProt Accession # Q8N0Z9 and Q86VR7), while IgC2 is a common feature of all members except for VSIG8 (UniProt Accession # P0DPA2) and TIGIT. Moreover, they are all type I transmembrane proteins with a wide range of expression. The structural composition of the extracellular domain (ECD) is highly conserved between hVSIG and mVSIG members.

Although the tissue distribution of VSIG proteins is not fully described due to antibody unavailability for some of the family members, genetic analysis and sequencing data reveal a diverse range of expression of VSIG proteins from testicular germ cells (VSIG1 and VSIG3) (32, 34) to the hair shaft (VSIG8) (35, 36) and immune cells (VSIG4 and TIGIT) (23–26, 33, 37–39), hence serving a wide array of functions in both humans and mice. A brief overview of VSIG family members and their functions, expression patterns, and roles in immunotherapy are summarized in **Table 1** and **Table 2** In the following context, we will discuss the role of each member of the VSIG family in detail, provide a comprehensive summary of our current understanding of these proteins, and highlight their potential as new targets for ICB therapy.

**Table 1:** Brief overview of VSIG family members.

	VSIG1	VSIG2	VSIG3	VSIG4	VSIG8	VSIG9	VSIG10	VSIG10 L
**Aliases**	GPA34	CTH, CTXL	BT-IgSF, IgSF11	VSIG4, Z39IG	C1orf204	TIGIT, VSTM3, WUCAM	–	–
**Cytogenic location**	Xq22.3	11q24.2	3q13.222	Xq12	1q23.2	3q13.31	12q24.23	19q13.41
**Exons**	10	7	12	8	7	6	11	10
**Discovery/First** **Report**	2006(30)	1996(19)	2002(32)	2000(29)	2006(36)	2009(26)	2015(140)	2016(144)
**Binding Partners**	–	–	VISTA	C3 (C3b),LTA, MS4A6D	VISTA	CD155, CD112, CD113	–	–
**Structure Of ECD**	IgV-IgC2	IgV-IgC2	IgV-IgC2	IgV-IgC	IgV-IgV	IgV	IgC2-IgC2-IgC2- IgC2	IgC2(type 1)-IgC2 (type 2)
**Length of amino acids (ECD domain) for Human & Mouse**	H:387 aa (211aa)	H:227 aa (220aa)	H:431 aa (219aa)	H:399 aa (264aa)	H:414 aa (242aa)	H:244 aa (120aa)	H:540 aa (383aa)	H:867 aa (749aa)
	M:407 aa (212aa)	M:328 aa (220aa)	M:428 aa (218aa)	M:280 aa (unreviewed)	M:417 aa (241aa)	M:249 aa (120aa)	M:558 aa (406aa)	M:868 aa (736aa)
**Uniprot ID**	Q86XK7	Q96IQ7	Q5DX21	Q9Y279	P0DPA2	Q495A1	Q8N0Z9	Q86VR7
**Similarity with mVSIG**	81% *	85% *	95% *	80% **	88% *	65% *, 77% ***	63% *	75% *

*Based on ECD, ** Based on IgV domain, *** Based on cytoplasmic region.

**Table 2:** Diverse expression and function of VSIG family members.

VSIG Members	Expression in Immune cells	Expression In Tissue	Expression in Cancer	Role in Immunotherapy
VSIG1	–	Stomach, testis, ovary, liver	Esophageal carcinomas, gastric cancer, Ovarian cancers(30,44,47) HCC(51) Colon cancer(47,52) Lung cancer(46)	–
VSIG2	Macrophage B cell	Colon, stomach, prostate, trachea, thyroid glands	AML (58); Colon adenocarcinoma(62) Pancreatic cancer(60) Lung adenocarcinoma(61)	Positively correlated with B cell and M1 macrophage infiltration (62)
VSIG3	–	Brain, testis	Colorectal cancer, hepatocellular carcinomas, Gastric cancer (63,69) ; gliomas(70)	Negative regulation of T cell activation(21,22,67)
VSIG4	Tissue resident macrophage	Liver, peritoneum, Pancreas, colon	Lung cancer (93); Breast cancer (94) Ovarian cancer (95) Multiple myeloma (MM) (96) High-grade glioma (97)	Negative regulation of Tcell activation(23,73,74,92)
VSIG8	–	Oral epithelium, hair shaft & follicle, nail matrix	Head and neck cancer(#) Thyroid cancer(#) Colorectal cancers(#) …	Negative regulation of T cell activation(99,101);
TIGIT	T cell, NK cell, Treg	Lymphoid tissue	Melanoma, NSCLC, Colorectal cancer,HCC, AML, Glioblastoma(124-128,137) …	Negative regulator of immune cells(26,107,131,135…)
VSIG10	DC	Intestinal epithelium	Adenocarcinoma (141)	Negative regulation of CD4+ T cell activation(*)
VSIG10L	–	Saliva gland, oesophagus	Lung squamous cell carcinoma (145)	–

(*Reference from US patent (Application #20200270343).

(#)Reference from THE HUMAN PROTEIN ATLAS.

Website: https://www.proteinatlas.org/ENSG00000243284-VSIG8/pathology.

## VSIG1

IgSF is a large protein superfamily of cell surface and soluble proteins involved in adhesion processes, binding, and cell recognition. Members of this superfamily are defined by structural similarities to immunoglobulins and the presence of an Ig domain (40). VSIG1 is a typical IgSF with two extracellular Ig-like domains and a short cytoplasmic domain. It is also known as the radioiodinated cell surface A33 antigen or glycoprotein A34, which was first characterized to be a tissue-restricted cell surface protein predominantly expressed in the gastric mucosa (30, 41). It was subcellularly localized in the adherens junctions of glandular epithelia and was critical for ensuring proper differentiation of glandular gastric epithelium (41). Three alternatively spliced isoforms of VSIG1, including VSIG1A, B and C, were identified in mice, with the latter being specifically expressed in the testis (41). In this tissue, VSIG1 was found to be a ZO1 (zonula occludens-1)-binding junctional adhesion molecule (JAM) localized on the surface of sperm cells to facilitate their interactions with Sertoli cells, suggesting that VSIG1 may be involved in supporting spermatogenesis (34). However, this has been challenged by a recent report showing that *VSIG1* knockout mice had normal development and function of sperm cells, and whether the absence of phenotype upon *VSIG1* deletion was caused by unknown compensatory mechanisms or genetic redundancy remains to be investigated (42).

Due to its abundant expression in the stomach, VSIG1 has been extensively studied in gastric cancers. In a cohort of 232 gastric adenocarcinoma samples, Chen et al. reported that VSIG1 was significantly reduced at both the mRNA and protein levels in gastric tumor tissues compared to paired noncancerous gastric mucosal tissues (43). Inoue et al. also reported a dramatic decrease in VSIG1 expression in 219 of 362 gastric cancer specimens (44). Furthermore, downregulation of VSIG1 was significantly correlated with poor overall survival and worse clinical outcome in gastric cancer patients (43–45), suggesting that *VSIG1* may function as a tumor suppressor gene. In support of this, overexpression of VSIG1 diminished the proliferation and migration of multiple gastric cancer cell lines *in vitro (* 44).

In contrast, VSIG1 seemed to be upregulated in a variety of nongastric carcinomas (30, 44, 46, 47). It was identified as a signature gene for gastric-type differentiation of serrated pathway-associated colon carcinoma (47–49) and lung adenocarcinoma (50). The coexpression of VSIG1 in the cytoplasm of hepatocytes with thyroid transcription factor 1 (TTF1) was also considered to be a potential lineage shift indicator of conventional to gastric-type hepatocellular carcinoma (HCC) (51). In the same study, Gurzu et al. further revealed that VSIG1 was strongly correlated with epithelial-mesenchymal transition (EMT) genes, such as E-cadherin and N-cadherin and VIM (51). Since VSIG1 is known to function as a JAM involved in tight junction assembly, these data implicated a potential role of VSIG1 in modulating EMT during tumor metastasis. In support of this, Bernal et al. showed that VSIG1 knockdown increased while gain of VSIG1 inhibited the migration of colon cancer cells (52). Overall, although the function of VSIG1 in modulating antitumor immune responses has not been explored thus far, given its importance as a cell surface tumor suppressor in contraining tumor growth and metastasis, targeting VSIG1 would be of great value for the treatment of different types of cancer (30).

## VSIG2

VSIG2 is composed of an ECD of 220 aa containing IgC2 and IgV domains and a cytoplasmic tail of 63 aa (28). VSIG2 is also known as CTXL (cortical thymocyte-like protein). It was initially identified as a marker predominantly expressed on cortical thymocytes in *Xenopus* and was designated CTX (cortical thymocyte of Xenopus). Due to the abundant expression of VSIG2 on double-positive thymocytes of Xenopus and on recent T-cell immigrants in chickens, it was considered to be involved in T-cell development in these species (27). However, by cloning its mouse and human homologues, namely, CTM (cortical thymocyte of mouse) and CTH (crotical thymocyte of human), respectively, VSIG2 was found to be abundantly expressed in the thyroid glands, trachea, prostate, colon, and stomach but weakly expressed in the lung and bladder but not in the thymus (28). These initial data suggest that VSIG2 may be an ancestral lymphocyte receptor before the introduction of somatic rearrangement in mammals.

Although the physiopathological function of VSIG2 remains to be explored, a close association of VSIG2 with the progression of various human diseases has been demonstrated in recent years using multiomics approaches, highlighting the potential of VSIG2 as a biomarker for the diagnosis of many diseases. VSIG2 was found to be significantly upregulated in the corneal samples of Fuchs endothelial corneal dystrophy (FECD) patients (53), in the intestinal biopsy of irritable bowel syndrome (IBS-D) patients (54), in the plasma of acute tubular injury and interstitial fibrosis/tubular atrophy patients (55), and in the plasma of incident heart failure patients (56). Moreover, the single nucleotide polymorphisms (SNPs) of VSIG2 are strongly associated with serologic profile and cytokine phenotype in systemic lupus erythematosus (SLE) (57). Aberrant VSIG2 expression was also found in tumors. Heimeng et al. reported that VSIG2 expression in acute myeloid leukemia (AML) patients correlated with poor prognosis according to The Cancer Genome Atlas (TCGA) and Gene Expression Omnibus (GEO) databases (58). VSIG2 expression has been found to be significantly upregulated in patients with nonmuscle invasive bladder cancer but downregulated in patients with muscle invasive bladder cancer; thus, it could serve as a biomarker of invasiveness in bladder cancers (59). In primary lung adenocarcinoma and pancreatic cancer, VSIG2 was characterized as a member of the DNA methylation-related prognostic signature (60, 61). High expression and methylation of VSIG2 correlated with poor survival in these cancer patients (58, 60, 61).

The potential effect of VSIG2 in modulating antitumor immunity was also implicated in colorectal cancers. In a recent study, Cui Z et al. reported lower expression of VSIG2 in colon adenocarcinoma (COAD) samples, and its downregulation was associated with a poor overall survival rate in COAD patients (62). However, it appears that *VSIG2* functions as a tumor suppressor gene to ensure tumor immune surveillance rather than an immune checkpoint molecule in this particular cancer type. Interestingly, VSIG2 expression positively correlated with B-cell and M1 macrophage infiltration (62). Since these cells are normally the most abundant antigen-presenting cells in tumor tissues, dissecting the role of VSIG2 in these cells may have implications for understanding the biology of T-cell activation in the tumor microenvironment.

## VSIG3

VSIG3 contains a V-type and C2-type immunoglobulin domain, a C-terminal PDZ domain, and a transmembrane domain (63). It was first cloned in 2002 and was then indicated to be a cell adhesion molecule that mediates homophilic cellular interactions (32). *VSIG3* is also known as IgSF gene 11 (IgSF11) or brain- and testis-specific IgSF (BT-IgSF) because of its abundant expression in these two organs in mammals (32, 64). hVSIG3 contains 12 exons encoding two isoforms that share 97% amino acid identity and are thus considered to be identical in function. VSIG3-mediated cell adhesion can regulate the development of neurons and excitatory synaptic transmission and the differentiation of osteoclasts, and its mutation in zebrafish was associated with impaired migration and survival of melanophores (65, 66).

In addition to its role in cell adhesion, one of the most notable functions of VSIG3 relies on its capacity to regulate immune responses. VSIG3 is reported to be a ligand for the novel B7 family immune checkpoint V-domain immunoglobulin suppressor of T-cell activation (VISTA) (21, 22, 67). VISTA is mainly expressed on naïve T cells and functions as an important regulator in maintaining T-cell tolerance through the induction of peripheral T-cell deletion (68). Truncated VSIG3 ECD containing either the IgV- or IgC2-type domain bound to human VISTA protein in a similar manner as the full-length ECD. Most importantly, the crosslinking of VSIG3 during TCR stimulation significantly inhibited T-cell activation by reducing the production of cytokines and chemokines. Blockade of VISTA significantly attenuated VSIG3-mediated T-cell inhibition, suggesting that this process is dependent on its recognition with VISTA (67). The VSIG3 and VISTA interaction was further demonstrated by Xie et al. using a coimmunoprecipitation (Co-IP) assay (21). They also revealed the crystal structure of the human VSIG3 ECD and designed a small molecule inhibitor, K284-3046, based on protein−protein docking analysis. This chemical inhibitor showed potent effects in diminishing VSIG3-mediated T-cell suppression (21).

The identification of VSIG3 as a binding partner for VISTA has important implications for tumor immunotherapy. As a well-known coinhibitory molecule for T cells, VISTA is highly expressed in myeloid cells and T cells that infiltrate into tumors and help create an immunosuppressive tumor microenvironment by enhancing Treg differentiation and inhibiting T-cell activation. In contrast, VSIG3 was highly expressed in a number of cancers, such as colorectal cancers, gastric cancer, hepatocellular carcinomas, and gliomas, but not on immune cells (63, 69). Suppression of VSIG3 by small interfering RNA (siRNA) attenuated the proliferation of gastric cancer cells *in vitro*, suggesting that the expression level of VSIG3 is essential for the fate of cancer cells (63). Ghouzlani et al. also found that high VSIG3 expression was related to a strong immunosuppressive microenvironment and functionally compromised T cells in glioma (70). Therefore, it is speculated that highly upregulated VSIG3 in tumor cells could reinforce immune inhibitory signals to VISTA-expressing T cells in the tumor microenvironment, generating antibodies or chemical inhibitors that specifically block the VSIG3-VISTA interaction and could increase the efficiency of VISTA-based ICB therapy (22).

## VSIG4

*VSIG4*, also known as Z39Ig or CRIg, was first described in 2000 as an X chromosome-located gene (29). VSIG4 contains two Ig-like domains, one complete IgV domain and a truncated IgC domain, and shares all the conserved amino acids with known B7 family members; thus, it is also considered a B7 family-related protein (23). There exist two alternatively spliced forms of human VSIG4 protein: the long isoform of VSIG4(L) encodes both IgV and IgC2 domains, while the short isoform of VSIG4(S) encodes only a single IgV domain (37). In comparison, murine VSIG4 only shows the short isoform of a single IgV domain. Human and mouse VSIG4 share 83% sequence homology within the IgV domain (71). The IgV domain is responsible for binding to the β chain of C3b to promote phagocytosis (72). The intracellular portion of VSIG4 contains a cAMP/cGMP-dependent protein kinase phosphorylation site and a protein kinase C phosphorylation site, yet the function of these sites remains unclear (29). Notably, VSIG4 expression is restricted to tissue resident macrophages, including liver Kupffer cells (37), peritoneal macrophages (23), pancreatic macrophages (73, 74), synovial lining macrophages in the joint, and interstitial macrophages in the heart (38, 75, 76); however, the extent of expression on these macrophages can be downregulated by inflammatory cytokines (77, 78). This expression pattern suggests a role for VSIG4 in maintaining tissue homeostasis.

VSIG4 is well known as the complement receptor of the Ig superfamily (CRIg) with a high binding affinity to complement components C3b and iC3b. Upon activation, C3—the central component of the complement system—is cleaved to a small C3a fragment and a large C3b fragment by C3 convertase, and iC3b is subsequently produced by complement factor I-mediated cleavage of C3b (79, 80). Both C3b and iC3b are potent opsonins that can coat the surface of invading pathogens, apoptotic cells, or immune complexes to facilitate their clearance by the mononuclear phagocytic system. By recognizing C3b- and iC3b-tagged pathogens, accumulating evidence suggests a critical role of VSIG4 in host defense against bloodstream infections by promoting Kupffer cells to take up complement-tagged bacteria, fungi, and viruses (37, 81, 82). This function is vital to prevent the dissemination of pathogens to some vulnerable organs, such as the heart and kidney (37). However, it was also reported that VSIG4 facilitated a relatively slow clearance of circulating bacteria when compared to scavenger receptor-mediated fast clearance (83). This slow clearance of circulating bacteria may be essential to enable a timely induction of adaptive immune responses. Moreover, VSIG4 was suggested to be a pattern recognition receptor that directly binds and captures blood-borne gram-positive bacteria by recognizing lipoteichoic acid (LTA) during *Staphylococcus aureus* infection (84).

Structural analysis revealed that VSIG4 can also dramatically inhibit the activity of C3 and C5 convertase upon binding to C3b, thereby preventing the alternative pathway of complement activation (72). Given that inappropriate activation of complement is usually associated with unwanted and exacerbated inflammation, recombinant VSIG4-Fc protein has been exploited as a decoy receptor to efficiently alleviate a variety of inflammatory diseases by preventing excessive complement activation, such as experimental autoimmune uveoretinitis (EAU) (85), intestinal ischemia/reperfusion (IR) injury (86), type 1 diabetes (73, 74), arthritis (71, 72), and SLE (87). This complement inhibitory function could be further improved by fusing VSIG4 with the alternative pathway inhibitory domain of factor H (FH) (88). In addition, recent studies reported that VSIG4 can directly inhibit macrophage-mediated inflammation independent of complement. Huang et al. found that macrophages lacking VSIG4 showed increased activation of the NLRP3 inflammasome upon stimulation (89). VSIG4 was found to interact with the transmembrane protein MS4A6D to form a surface inhibitory signaling complex, leading to attenuated NLRP3 inflammasome activation *via* a JAK2-STAT3-A20 signaling cascade (89, 90). In addition, VSIG4 was also able to intervene in mitochondrial pyruvate metabolism in macrophages by activating the PI3K-Akt-STAT3 pathway, thereby resulting in reduced oxidative phosphorylation and diminished M1 polarization of macrophages (91). These data suggest the possible applications of targeting VSIG4 in treating inflammatory diseases.

**Figure 1:**
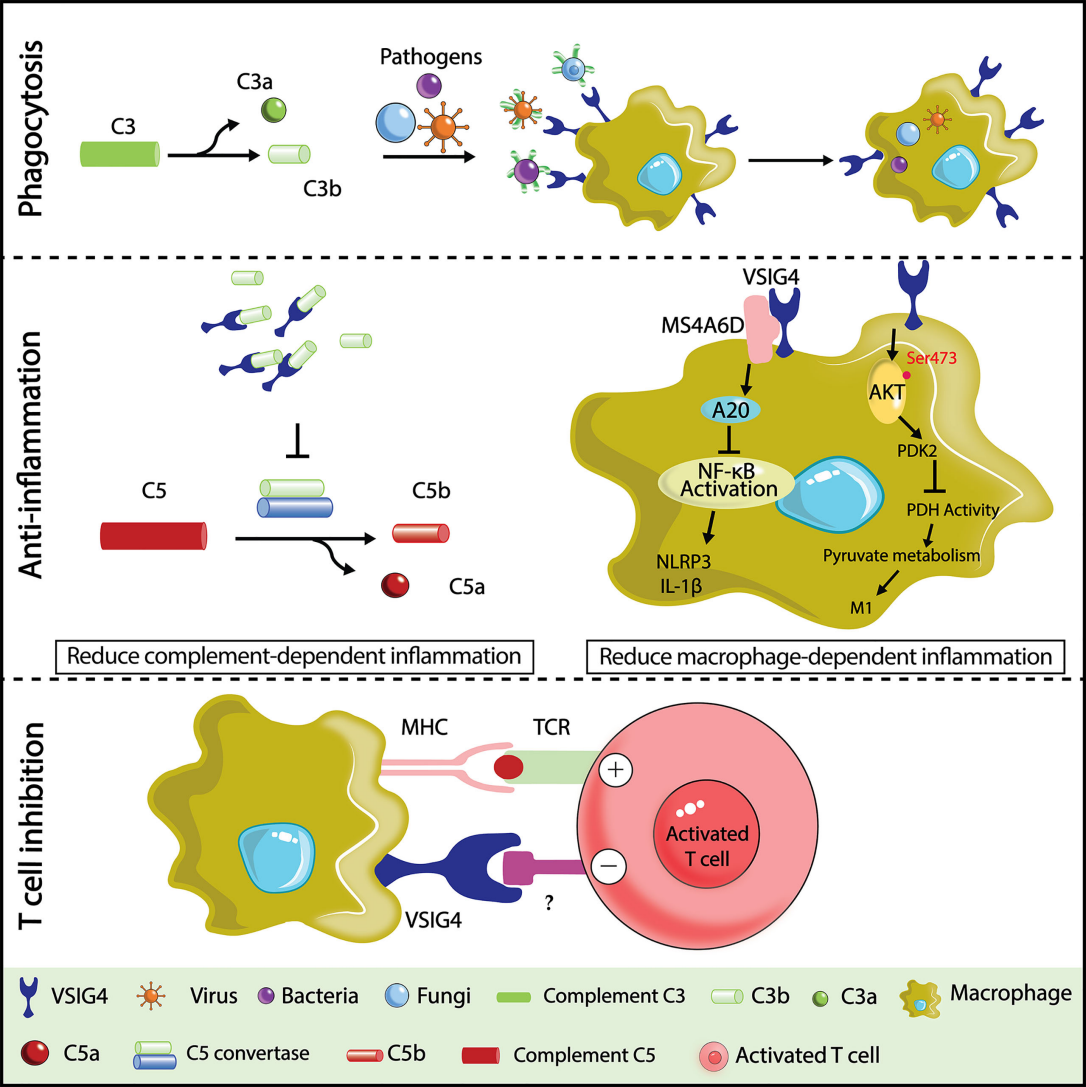
Illustration of VSIG4 functions. In host defense against bloodstream infections,VSIG4 recognizes C3b and helps macrophages phagocytose C3b- or iC3b-tagged pathogens (bacteria, viruses, fungi, etc.). In a variety of complement activation-dependent inflammatory diseases, VSIG4 delivers anti-inflammatory signals by binding C3b to prevent the alternative pathway of complement activation. VSIG4 also inhibits macrophage M1 activation by regulating inflammasome activation and pyruvate metabolism. VSIG4 also inhibits T-cell activation, proliferation, and IL-2 production upon binding to an unknown ligand on T cells.

As a macrophage-specific immune regulator, VSIG4 is a potent coinhibitory ligand that strongly suppresses T-cell proliferation and cytokine production. T-cell stimulation in the presence of recombinant VSIG4 caused T-cell anergy, cell cycle arrest at the G0/G1 phase (23, 73, 74, 92), and skewed differentiation of CD4^+^ T cells towards Foxp3^+^ Treg cells. Importantly, this T-cell inhibitory effect was only found with plate-bound but not soluble VSIG4 protein, suggesting that the crosslink of VSIG4 with a putative binding partner on the surface of T cells is required to deliver inhibitory signals. Indeed, VSIG4 can directly bind activated T cells without the need for serum, demonstrating the existence of a complement-independent ligand of VSIG4 on T cells, which remains to be determined. Although VSIG4 expression is restricted in tissue-resident macrophages at a steady state, several studies have reported an upregulation of VSIG4 expression in lung cancer (93), breast cancer (94), ovarian cancer (95), and multiple myeloma (MM) (96). By examining VSIG4 expression in tumor tissues, it was found to be highly enriched in tumor-associated macrophages but not in tumor cells or other immune cells (93). High expression of VSIG4 is correlated with poor prognosis of high-grade glioma (97), and its deficiency led to significantly inhibited growth of Lewis lung cancer cells (LLC) in mice (93). Based on these findings, VSIG4 is becoming an attractive macrophage-specific immune checkpoint molecule in cancer immunotherapy. Identifying the ligand of VSIG4 on T cells would be pivotal for understanding the mechanisms whereby VSIG4 modulates antitumor T-cell responses and is fundamentally important for developing high-efficacy inhibitors that aim to block VSIG4-mediated T-cell suppression in cancer **Figure 1**.

## VSIG8

VSIG8 is a relatively less explored member of the VSIG family, approximately 45 kDa in size. Mature hVSIG8 contains two Ig-V domains, which are a part of the ECD spanning 242 aa. hVSIG8 shares 88% and 89% identity with the VSIG8 of mouse and rat, respectively. It was identified through proteomic analysis of the human hair shaft (35, 36) and found to also be expressed in the oral epithelium, superficial layers of the nail matrix, and hair follicles (98). Interestingly, Wang et al. reported that immobilized recombinant VSIG8 could suppress human T-cell proliferation and cytokine production and decrease the differentiation of naïve CD4^+^ T cells towards Th1 cells, confirming its role as a negative regulator of T-cell responses (99). VSIG8 was later reported to be a putative binding partner of VISTA (100), and a US patent (WO2016090347 A1) also reported the interaction of VSIG8 and VISTA, demonstrating the suppressive effects of this interaction on T cells. In addition, Chen et al. demonstrated the VSIG8-VISTA interaction by ELISA, MST and Co-IP assays and confirmed its function in inhibiting human T-cell activation (101). However, a recent study using a functional ELISA suggested no interaction between human VSIG8 and VISTA (67). Similarly, George et al. generated a two-sided fusion protein that contained the ECD of both VSIG8 and OX40 L and reported no binding between this fusion protein and recombinant VISTA, although it was able to bind VISTA-expressing macrophages or tumor cells. Nevertheless, this VSIG8-Fc-OX40 L fusion protein stimulated T-cell activation and antitumor activity, possibly by blocking VSIG8-mediated inhibitory signaling (102). Future studies generating VSIG8-deficient animals and blocking antibodies will further enhance our understanding of this potential immune checkpoint molecule in cancer immunotherapy.

## VSIG9

VSIG9, well known as TIGIT, with a full name of T-cell immunoglobulin and ITIM domain (also known as WUCAM or Vstm3), is currently one of the most attractive and promising immune checkpoint targets. TIGIT also belongs to the poliovirus receptor (PVR)/nectin family and is widely expressed on activated NK cells, CD8^+^ T cells, CD4^+^ T cells, and Treg cells (24–26, 33, 39). TIGIT was discovered in 2009 by three independent groups (25, 26, 33). One reported that TIGIT was an adhesion molecule mediating TFH (follicular T helper) and FDC (follicular DC) interactions (33), whereas the other two identified TIGIT as a coinhibitory receptor on T and NK cells (25, 26). The structure of TIGIT comprises a short intracellular domain with one immunoglobulin tyrosine tail (ITT)-like motif and one immunoreceptor tyrosine-based inhibitory motif (ITIM), a type I transmembrane domain, and an extracellular IgV domain (26, 33, 39). While there is a 58% overall sequence identity between human and mouse TIGIT (25, 26), their ITIM-containing sequences that confer immune inhibitory functions are identical. TIGIT has three ligands, including CD155, CD112, and CD113, which all belong to the PVR/nectin family receptors (25, 103, 104). Their expression features and binding affinity with TIGIT are listed in **Table 3**.

**Table 3:** Expression patterns of TIGIT ligands and their relative binding affinity and function.

	CD155	CD112	CD113
**Expression cell types**	DCs, T cells, B cells, macrophages, tumor cells	DCs, T cells and B cells, CD14+ cells, monocyte, tumor cells	T cells, tumor cells
**Receptors and Binding Affinity(+)**	TIGIT (++) CD226(+) CD96(+)	TIGIT (+) CD226 (+) CD112R (++)	TIGIT (+)
**Function**	Inhibitory ligand	Inhibitory ligand	Inhibitory ligand

**Figure 2:**
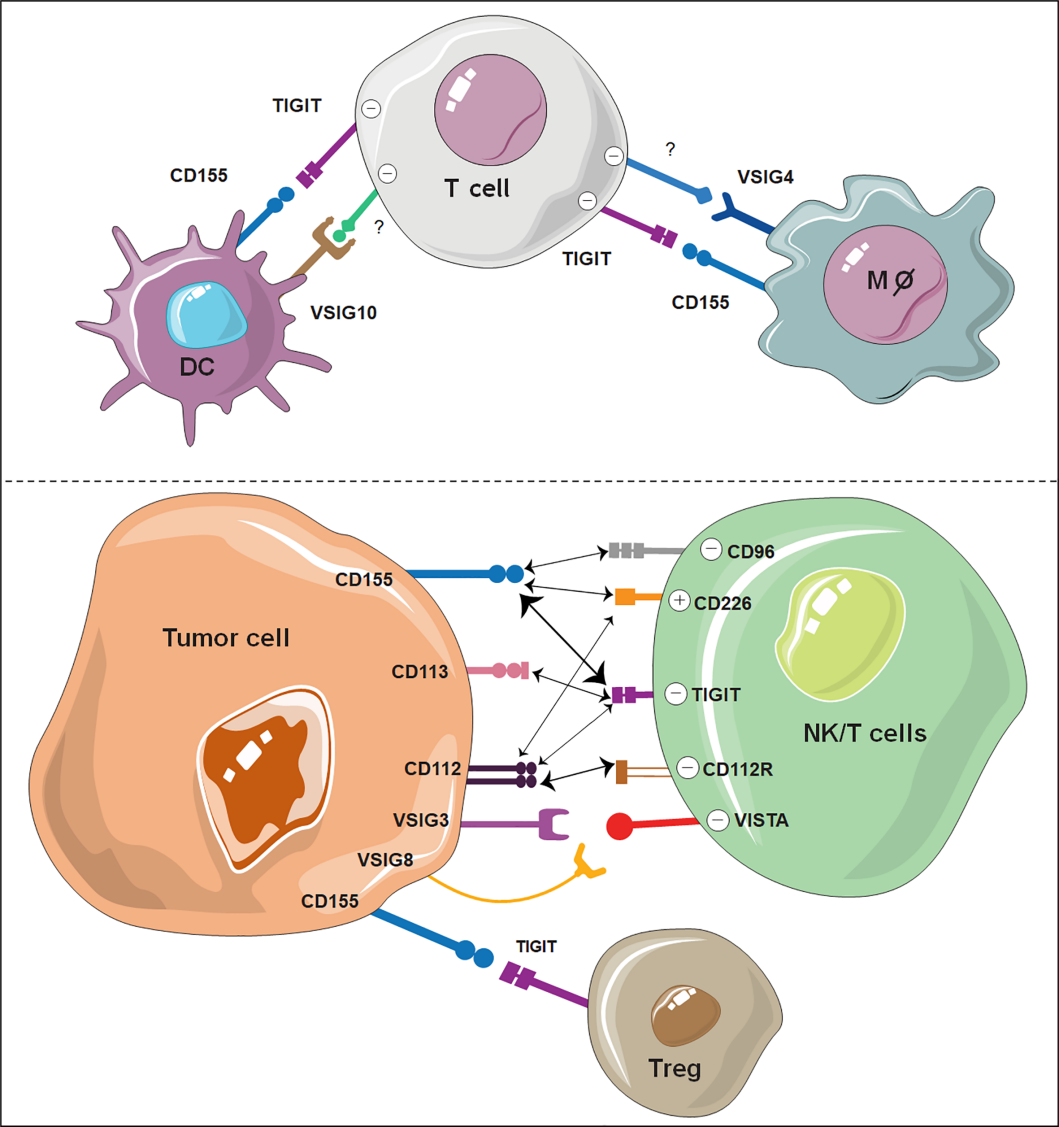
Illustration of VSIG family members as potential ICBs on immune cells. The interaction of TIGIT, VSIG3, VSIG8 and VSIG4 with other ligand-expressing cells shows great potential as novel immune checkpoints. VSIG10 also shows potential as a coinhibitory receptor on DCs. The width of the arrows is proportional to the relative binding affinities. The strongest interactions are between TIGIT/CD155 and CD112R/CD112. Negative (-) represents an inhibitory signal, and positive (+) represents an activating signal.

Among these ligands, CD155 exhibited the highest affinity with TIGIT (104, 105). CD155 is mainly expressed on dendritic cells (DCs), T cells, B cells and macrophages. Engaging of TIGIT with CD155 has been shown to prevent excessive immune cell activation and sustain immune homeostasis (33, 106–108). Notably, there are two other PVR family receptors, CD226 and CD96, which share sequence homology with TIGIT and compete with TIGIT for CD155 binding (109–111). However, as opposed to TIGIT and CD96, CD226 acts as an activating receptor that promotes T-cell and NK-cell activation opon CD155 ligation (112–114). TIGIT binds CD112 and CD113 with lower affinity than CD155, and the functional consequences of their binding have been less characterized. CD112 is also known as the ligand for the coinhibitory receptor CD112R, which was recently discovered as an immune checkpoint receptor expressed on T cells and NK cells (109, 115). Similar to CD155, CD112, another common ligand of TIGIT, can also bind CD226 (109). The competition and balance among TIGIT, CD226, CD96, and CD112R for the same ligands is quite complex and has been extensively reviewed elsewhere (112, 116, 117). The interactions between TIGIT and its ligands with other VSIG members are summarized in **Figure 2**.

Because of the binding affinity advantage, the TIGIT-CD155 interaction prevails in TIGIT-mediated immune inhibition (26). The TIGIT-CD155 axis exerts its inhibitory effects on T and NK cells through three distinct mechanisms of action, including 1) a cell intrinsic manner by transmitting inhibitory signals to the effector cells (26); 2) a cell extrinsic manner by modulating the cytokine profile of CD155-expressing cells, such as DCs and macrophages (26, 107); and 3) by competing with CD226-mediated costimulatory signals (118). The cell intrinsic effect of TIGIT was well characterized by Inozume et al. by expressing truncated CD155 without a cytoplasmic domain, which was sufficient to suppress T-cell production of IFN-γ in a TIGIT-dependent manner (119). Similarly, agonistic anti-TIGIT mAbs were capable of dampening mouse and human T-cell proliferation and cytokine production (39, 118, 120). Following TIGIT-CD155 binding, the ITT-like motif is phosphorylated and subsequently bound to growth factor receptor-bound protein 2 (Grb2) or β-arrestin 2, which leads to the recruitment of SHIP-1 and SHP2 and abolishment of PI3K and MAPK and NF-κB signaling (121). TIGIT can also work in a cell-extrinsic manner by modulating the cytokine profile of CD155-expressing cells, such as DCs and macrophages. The altered cytokine milieu, e.g., increased IL-10 levels and decreased IL-12 levels, in turn, can lead to attenuated activation of NK and T cells (26).

The tumor microenvironment has taken advantage of the TIGIT-CD155 inhibitory pathway as an important strategy to evade immune surveillance and thus result in uncontrolled tumor growth (122, 123). TIGIT is highly expressed on CD8^+^ tumor-infiltrating lymphocytes (TILs) in various tumors, such as gastric cancer, colon cancer, breast cancer, melanoma, and NSCLC (124–127). TIGIT^+^ CD8^+^ TILs are dysfunctional with reduced effector cytokine production and impaired degranulation, exhibiting a typical feature of exhaustion. Blocking the TIGIT pathway can drastically reverse T-cell exhaustion. In AML, TIGIT^+^ CD8^+^ exhausted T cells were reinvigorated by knockdown of TIGIT expression (128). The prominent advantage of TIGIT over other immune checkpoints lies in its potent ability to restrain NK-cell responses. Upon binding to its ligand CD155 expressed by tumor cells, TIGIT-expressing NK cells dramatically diminish their cell cytotoxicity (129, 130). Zhang Q et al. showed that antibody blockade of TIGIT prevented NK exhaustion and unleashed antitumor immunity in an NK-cell-dependent manner, collectively leading to tumor regression (131). Moreover, TIGIT is highly expressed on Treg cells and is essential to maintain the suppressive capabilities of Tregs, which potentially inhibit a variety of immune cells by suppressing Th1 and Th17 cells (132–135). A study demonstrated that TIGIT suppresses antitumor immunity primarily *via* Tregs but not CD8^+^ T cells (135). In addition, TIGIT also functions as a ligand to skew the maturation or polarization of intratumoral myeloid cells, including DCs and macrophages, towards a state with increased IL-10 but decreased IL-12 secretion (26). This results in DC tolerance and alternative activation of macrophages, both contributing to tumor immune tolerance.

Owing to the importance of TIGIT-CD155 engagement in NK and T-cell dysfunction in tumors, developing therapeutic agents to block this pathway holds great promise for cancer immunotherapy. There is strong evidence that TIGIT blockade has a direct effect in reversing T-cell dysfunction in cancer patients. Anti-TIGIT mAb treatment has been shown to escalate the proliferation, cytokine production, and degranulation of bone marrow CD8^+^ T cells from MM patients and peripheral blood CD8^+^ T cells from melanoma patients (125, 136). Recent advances have also proposed a dual blockade of PD-1 and TIGIT as a more inspiring method for cancer immunotherapy than a single TIGIT blockade. Whereas blocking each of the PD-1/PD-L1 or TIGIT pathways does not remarkably impede the growth of CT26 tumors, a dual blockade synergizes to increase the proliferation and function of antitumor CD8^+^ T cells, which results in protective memory T cells, complete tumor rejection, and overall prolonged survival (126, 131). These effects have been abrogated upon CD8^+^ T-cell deficiency. The translational potential of dual PD-1/TIGIT inhibition has already been demonstrated; it increases the proliferation and function of intratumoral antigen-specific CD8^+^ T cells in melanoma patients to an extent that is much more dramatic than a single blockade (119, 125). A recent phase II study also indicated that dual PD-L1/TIGIT blockade (atezolizumab/tiragolumab) has superior clinical benefits compared to PD-L1 blockade alone in NSCLC patients, despite similar profiles of toxicity and tolerability (137). Apart from PD-1, TIGIT blockade could also synergize with other ICBs in cancer immunotherapy. For instance, TIGIT and TIM-3 inhibition in mice cooperated to promote an antitumor immune response (135); dual blockade of TIGIT and LAG3 improved the treatment efficacy in a mouse model of anti-PD-1-resistant lung cancer (138). In conclusion, TIGIT, as a new immune checkpoint, possesses great potential for cancer immunotherapy. Effective tumor control for certain types of cancer can be expected by combining anti-TIGIT with other ICB inhibitors.

## VSIG10 and VSIG10 L

VSIG10 contains four Ig-like C2-type domains in its ECD with 63% identity between hVSIG10 and mVSIG10 (139, 140). VSIG10 was highly expressed on both normal and cancer epithelial cells based on transcriptional data. Moreover, Papasotiriou et al. reported the overexpression of VSIG10 in adenocarcinoma; however, no expression was observed in melanoma, prostate, breast, or pancreatic cancer (141). Until recently, there was no report about its biological function. According to a US patent (Application #20200270343), recombinant VSIG10-Fc fusion protein was able to suppress CD4^+^ T-cell activation and cytokine production, pinpointing its potential as an immune checkpoint inhibitor. Interestingly, VSIG10 was also predicted to be abundantly expressed by DC subsets both in humans and mice. Growing data points toward the importance of immune checkpoints expressed on DCs in dampening the antitumor response. For instance, DCs highly express PD-L1 and have been demonstrated to be an important target of PD-L1 blocking antibodies (142). These PD-L1-expressing DCs are identified to attenuate T-cell activation and regulate its response to ICBs (143).

Hence, the generation of anti-VSIG10 antibodies, as reported in the patent, may present a promising DC-targeting ICB cancer immunotherapy. Similar to VSIG10, VSIG10 L also contains IgC2 in its ECD. VSIG10 L is normally expressed in the healthy esophagus and squamous mucosa; however, it is downregulated in esophageal adenocarcinoma and Barrett’s esophagus (144). High expression is found in lung squamous cell carcinoma (145), pointing to the dual nature of VSIG10 L in cancers. Further exploration is needed, as they could be potential biomarkers or immune checkpoints.

## Conclusion

ICB treatment has brought a revolutionary advance for cancer therapy in the past decade. Antibodies targeting PD-1 and CTLA-4 were approved by the FDA and were proven to be effective against several cancer types. Despite tremendous success, over half of the patients remain poorly responsive to these regimens, possibly due to the involvement of multiple immune inhibitory pathways in the cancer microenvironment. Therefore, seeking new immune checkpoint molecules is becoming increasingly important for the optimization of ICB-based cancer immunotherapy. Here, we show that many VSIG family members show potent effects of T-cell inhibition in cancer, and antitumor immunity can dramatically benefit from the blockade of these molecules. The most attractive and promising member of the VSIG family is TIGIT, and its blockade has achieved great success in reinvigorating antitumor NK and T-cell responses (126, 131, 135). There are currently over 50 clinical trials underway to study the therapeutic effect, safety, and tolerability of TIGIT blockade in cancer, either using it alone or in combination with other cancer therapeutics. Apart from TIGIT, VSIG3, VSIG8, and VSIG4 also show great potential as novel immune checkpoints. As putative binding partners for the well-known coinhibitory molecule VISTA, both VSIG3 and VSIG8 were able to negatively regulate T-cell responses and can be targeted in certain cancer types in which antitumor immunity is predominantly affected by the VISTA pathway. VSIG4 is of particular interest because it is specifically expressed by tissue resident macrophages, which are becoming increasingly appreciated as critical contributors to tumor progression and metastasis. Blockade of VSIG4 to functionally reprogram macrophages thus stands out as an important complement to the current T-cell-based immunotherapy regimens. In addition, although not fully validated, VSIG10 shows potential as a coinhibitory receptor expressed by another important type of myeloid immune cell, namely, DCs, which are also largely overlooked in the field of immune checkpoint therapy. Overall, VSIG family proteins represent an important group of transmembrane receptors that emerge as immune checkpoints controlling the fates of multiple types of immune cells in tumors, spanning from myeloid cells to lymphoid cells. Therapeutically targeting these proteins could be beneficial to the current regimen of ICB treatment in cancer.

## Author Contributions

XZ and SK wrote the manuscript, LL and DH supervised the study and revised the manuscript. All authors contributed to the article and approved the submitted version.

## Funding

This work was supported by the NSFC (82003008), the China Postdoctoral Science Foundation (2021M703076 and 2020M682048), the Fundamental Research Funds for the Central Universities (WK9110000171 and WK9110000086), the Anhui Provincial Natural Science Foundation of China (2008085MH299) and the Postdoctoral Research Funding of Anhui Province in 2019 (2019B371).

## Conflict of interest

The authors declare that the research was conducted in the absence of any commercial or financial relationships that could be construed as a potential conflict of interest.

## Publisher’s note

All claims expressed in this article are solely those of the authors and do not necessarily represent those of their affiliated organizations, or those of the publisher, the editors and the reviewers. Any product that may be evaluated in this article, or claim that may be made by its manufacturer, is not guaranteed or endorsed by the publisher.

## Abbreviations

ICB, immune checkpoint blockade; CTLA-4, cytotoxic T-lymphocyte-associated protein 4; AML, acute myeloid leukemia; TCGA, The Cancer Genome Atlas; GEO, Gene Expression Omnibus; COAD, colon adenocarcinoma; VISTA, V-domain immunoglobulin suppressor of T-cell activation; Co-IP, coimmunoprecipitation; MM, multiple myeloma; TIGIT, T-cell immunoreceptor with immunoglobulin and ITIM domain; PVR, poliovirus receptor; TFH, follicular T helper; FDC, follicular DC; ITT, immunoglobulin tyrosine tail; ITIM, immunoreceptor tyrosine-based inhibitory motif; LTA, recognizing lipoteichoic acid; ELISA, Enzyme-linked Immuno Sorbent Assay; MST, Microscale Thermophoresis; ECD, extracellular domain; TTF1, subcellular localization of thyroid transcription factor 1; SLE, systemic lupus erythematosus; NSCLC, non-small cell lung carcinoma; FECD, fuchs endothelial corneal dystrophy; JAM, junctional adhesion molecule; SNP, single nucleotide polymorphisms; EMT, epithelial-mesenchymal transition; IBS-D, intestinal biopsy of irritable bowel syndrome; HCC, hepatocellular carcinoma.
